# Supplementary material for: Information Processing Speed Assessed with Letter Digit Substitution Test in Croatian Sample of Multiple Sclerosis Patients
Source: Diagnostics (Basel). 2022 Jan 4;12(1):111. doi: 10.3390/diagnostics12010111 (PMC8774378; doi:10.3390/diagnostics12010111)
Supplement: Supplementary file 1 [file diagnostics-12-00111-s001.zip › diagnostics-1510111-supplementary.pdf]

Table S1. LDST.

W	B	T	P	V	D	G	C	J
1	2	3	4	5	6	7	8	9

T	W	C	G	J	V	B	D	P	V	P	T	D	C	B

P	D	V	B	T	D	P	W	B	J	D	T	C	V	G

J	P	W	C	B	V	J	D	P	C	G	W	T	B	V

T	G	V	B	P	W	C	V	D	J	W	J	G	D	C

G	T	J	C	W	C	G	D	J	P	B	V	T	C	B

W	P	G	V	B	J	C	P	T	C	G	W	J	D	V

J	P	G	D	G	B	J	C	W	V	T	B	D	T	W

T	V	G	W	D	P	V	D	B	J	G	T	J	P	B

W	C	T	V	P	B	J	G	W	D	V	C	T	P	G
